# An Ovarian Arscess Containing a Lumbricoid Worm

**Published:** 1909-08

**Authors:** 


An Ovarian Abscess Containing a Lumbricoid Worm.—Dr. Henry D. Fry, of Washington, D. C., described this case, which occurred in a colored woman, who gave no history of nausea, vomiting, or lack of appetite before admission to the hospital. On admission she had a temperature of 103 degrees and a pulse of 132. There was a mass in her left side. After admission she vomited a lumbricoid worm, and examination of the stool showed that she passed eggs of the same worm. After the acute symptoms had subsided she was operated on, and the mass in the left side was found to be an abscess of the ovary. There were no adhesions to the intestines. The walls were thick everywhere except at one point, and there they were smooth and nonadherent. It is well known that the lumbricoid worms enter through the drinking water, travel over all mucous membrane surfaces communicating with the alimentary canal, and penetrate lumbar, psoas and abscesses. The female genital tract is also frequently involved, and the worms passed with the urine. Entrance may be gained by fistulous tracts to the intestine, or by passing over the perineum. A case of a lumbricoid worm in an ovarian abscess had been reported in which the worms were alive, but there were adhesions to the intestines.

In this case the worm was dead, flattened, six or seven centimeters long. It was identified by the pathologist. The author thought that in this case the worm had passed externally over the perineum into the vagina and then through the uterus and tube to the Graafian follicle.—*New York Medical Record.*

